# The Polyclinic

**Published:** 1907-04-20

**Authors:** 

**Affiliations:** Hon. Physician Marylebone General Dispensary


### Graduates' Medical Schools.

### THE POLYCLINIC.

### WHAT IT OFFERS TO THE GENERAL PRACTITIONER.

By COLONEL CLUTTERBUCK, M.D., M.R.C.P.Lond., Hon. Physician Marylebone General Dispensary.

Until a comparatively recent date, the opportunities available for qualified medical practitioners to keep themselves abreast of the times by attendance at systematised clinical demonstrations organised specifically on their behalf, were limited. A man could indeed go to one of the hospitals, and especially to the hospital with which he had been connected in his student days, and could expect, as a rule, to be received with courtesy and consideration, but he knew he had no *right* to be there. He could only see such of the routine work as happened to be in progress at the time of his visit; he could claim nothing beyond a recognition of the altruistic rule that no medical man should withhold from a professional brother information on any subject that can be of use in the treatment of disease. Besides the hospitals, there existed, of course, the medical societies: these were never intended to be mere schools for keeping up the knowledge of medical men in the ordinary business of medical life: they have work of a higher kind to do, the advancement of scientific knowledge and research. Moreover, their meetings can only be held at somewhat infrequent intervals during a limited part of the year, and so could only be available for those living in or near the town in which they existed. But many a medical man, and especially the busy general practitioner, wanted facilities more than such institutions could offer. He wanted to be able periodically, and at any season that might happen to be convenient to him, to attend at some place where, on payment of a reasonable fee, he could for himself see and learn how to apply the most recent methods of medical and surgical practice.

### How It was Started.

It thus became obvious that there was room for an institution devoted primarily to the instruction of qualified men, and more especially, perhaps, to the needs of the general practitioner. To meet this want the Polyclinic was established in the year 1899, its doors being open only to those holding a registrable medical qualification. Here we see the first advantage of such a teaching institution as compared with most previously existing bodies. A hospital or similar institution is, and must be, primarily devoted to the treatment of the sick, associated, of course, with the instruction, in certain recognised institutions, of medical students; the Polyclinic is primarily devoted to the needs of the qualified man, and adapts itself accordingly, thereby enormously increasing the value of medical treatment to the public generally. This does not mean that the treatment and needs of patients who happen to be seen at the Polyclinic are in any way ignored or neglected; on the contrary, such patients are either receiving treatment elsewhere from competent hands, while at the Polyclinic the proper methods, advantages, and results of such treatment are explained to attending members; or, on the other hand, the patients have the advantage of examination by, and the opinion of, some of the most highly-skilled and experienced members of the medical profession in London.

Before going further I would wish to explain that I do not for one moment mean it to be thought that there are not other institutions in London affording facilities to post-graduates on somewhat similar lines, and still less would I belittle the value of the admirable work that is being done at such institutions. I am here only indicating the value of the Polyclinic as it stands, merely remarking that it is not conducted on quite the same lines as are most, if not all, other post-graduate teaching institutions.

### Where It Is.

A point of importance in connection with such a college as the Polyclinic is its situation. The location of the college in Chenies Street may certainly be considered favourable. Situated near such important thoroughfares as Euston Road, Tottenham Court Road, and Gower Street, train, tube, tram, and 'bus are all available to those wishing to reach it.

### The Staff.

Since the main object of the college is teaching, pure and simple, it is obvious that in the selection of its regular staff regard must be had to their teaching capacity, apart from, and in addition to, their recognised skill and experience in the particular branches of the profession which they practise. This consideration cannot possibly enter so largely into the selection for appointments to a hospital staff. The value of a teacher in such a post-graduate institution is readily appraised by the members attending his lectures or classes; it is unlikely that a proved failure would remain long in a post where his failure was only too painfully evident. Hence every member joining for instruction has a sort of guarantee of competence on the part of the teachers to impart their knowledge in an assimilable form. Again, so much and such varied literature is now at the service of every medical man, that no teacher could afford to allow himself to get behind the times, at all events in his own particular line; a few awkward questions from intelligent members of his audience, many of them ready to verify for themselves his facts, would soon bring his shortcomings home to him. The relations of teacher and questioner here are very different from those that obtain between a teacher and a still unqualified student.

### Individual Teaching.

Another advantage is that it is fully recognised that a limitation in the numbers attending a practical class is all to the advantage of the learner, so that each one may reckon on more or less individual teaching; moreover, the instruction is given, whenever practicable, in a less formal manner than must be adopted in a course of purely systematic lectures. Nothing is more enlightening than intelligent questioning, or even reasonable arguments, on difficulties and doubts that come up during practical and clinical investigations and instruction; enlightening not only to the individual questioner, but to the other hearers, who thus see the different sides of the same facts as they present themselves to different minds. A certain "freedom of debate" is permissible in such circumstances.

Great importance is attached to making the teaching as useful as possible; in the practical classes, which can be attended for small fees, members are given every opportunity for seeing and making themselves proficient in the use of recent appliances and methods; for instance, directly the possibilities of the opsonin theory were recognised, a class was arranged at which members could see, and themselves assist, at the whole conduct of the process.

I remember once in my student days seeing one such practical demonstration. It was a case of tumour of the kidney, and the instructor, after going briefly but clearly through the conditions that might be present, eliminated them one by one, finally deciding upon renal tuberculosis. Some of the urine was centrifugalised, the deposit stained in the usual way for tubercle, and the bacillus there and then demonstrated under the microscope. Such a demonstration gave me, and, no doubt, all that attended it, a clearer grasp of the whole subject than any purely academic lecture or individual reading could have done. It is this style of teaching, applied, of course, to newer and ever-increasing numbers of methods, that can be expected at the Polyclinic. Further, members may for a small fee have a bacteriological, or other microscopical examination made of fluids or tissues, and, if they wish, can have it arranged so that they may be present and see the whole process carried out themselves. This offers great facilities for members renewing their acquaintance with laboratory technique, or for learning the application of new discoveries. New work is constantly being brought forward, being limited practically only by the demand which is shown to exist for the acquisition of information about it.

It is true the college is not affiliated to any hospital or other institution having beds, but even this may not be without a compensating advantage. In a hospital all the cases have to be gone through as they come—I refer chiefly to the out-patient department—and many may have to be attended to which are necessarily without much interest to a post-graduate. The patients brought up at the cliniques in Chenies Street, whether at practical classes or at the afternoon consultations, may all be expected to show some points of interest, having been selected to illustrate or enforce some point in diagnosis or treatment; or they have been brought by a member so that he and the patient may have the benefit of the opinion and advice of some special authority. As I have myself seen, the opinion of leading consultants in London is at the service of members in this way.

### The Conditions of Membership.

The institution is sufficiently progressive—in, I hope I may say, a favourable sense—to admit qualified women to the privileges of membership. Tutorial classes, or, as they may be described, coaching classes, are established periodically for those preparing to enter examinations for the higher degrees and diplomas. These classes are conducted by men with every qualification to instruct in the branches they deal with. I am able from personal experience to testify to the value of this tutorial teaching. Discoveries and theories are nowadays produced at such a rapid rate that it is impossible to avoid falling behind unless one has for some reason to study and appreciate each discovery of value as it appears, and to prove its efficacy in the amelioration of the conditions of the sick. Within the past ten years I have heard men who had attained to some eminence in the course of their practice ignore the usefulness of the microscope, and argue that the *x*-rays could teach a competent man nothing that he could not equally well discover by his own unaided senses of sight, hearing and touch. This attitude is one in which all men working in a groove must fear, to some extent, to fall, but it is a fatal one for the modern medical man.

### Its Social Side.

There are reading-rooms, a library, and museum provided for the use of members, current literature also being available for their use. A library and reading-room are conveniences that must be provided in any modern building, but they are of quite secondary importance in the objects of the college.

In order that graduates may, if they wish, gain an insight into the work and methods of the college before definitely committing themselves to membership, complimentary tickets are issued on application, entitling the holder to attend the afternoon consultations and systematic lectures for three successive days: everyone is thus enabled to form, from personal observation, an opinion as to the possible value of the institution to himself.

Most visitors to London, especially members of a profession, many of whom spent a considerable portion of their student life at a metropolitan medical school or hospital, look forward to meeting old friends and acquaintances, while those settled in London welcome an opportunity of greeting old professional comrades whose lot has taken them to the country, or abroad. The annual dinner held in connection with the Polyclinic affords a pleasant opportunity for the renewal of such acquaintanceships, when friends can discuss the affairs of the State, and learn how each has been faring since the last meeting—none the less prosperously, I'll be bound, because they have been persuaded at some time to become members of, and participate in the advantages offered by, the Polyclinic.

